# The progress test as a structuring initiative for programmatic assessment

**DOI:** 10.1186/s12909-024-05537-5

**Published:** 2024-05-21

**Authors:** Débora Cristina Alavarce, Melyssa Lima de Medeiros, Danylo de Araújo Viana, Flávia Abade, Joaquim Edson Vieira, José Lúcio Martins Machado, Carlos Fernando Collares

**Affiliations:** 1 Inspirali Educação, Belo Horizonte, MG Brasil; 2 Universidade Potiguar, Natal, RN Brasil; 3 Faculdade de Medicina, Universidade de São Paulo, São Paulo, SP Brasil

**Keywords:** Education, Medical, Undergraduate, Educational Measurement, Curriculum

## Abstract

**Background:**

The Progress Test is an individual assessment applied to all students at the same time and on a regular basis. The test was structured in the medical undergraduate education of a conglomerate of schools to structure a programmatic assessment integrated into teaching. This paper presents the results of four serial applications of the progress test and the feedback method to students.

**Methods:**

This assessment comprises 120 items offered online by means of a personal password. Items are authored by faculty, peer-reviewed, and approved by a committee of experts. The items are classified by five major areas, by topics used by the National Board of Medical Examiners and by medical specialties related to a national Unified Health System. The correction uses the Item Response Theory with analysis by the “Rasch” model that considers the difficulty of the item.

**Results:**

Student participation increased along the four editions of the tests, considering the number of enrollments. The median performances increased in the comparisons among the sequential years in all tests, except for test1 – the first test offered to schools. Between subsequent years of education, 2nd-1st; 4th-3rd and 5th-4th there was an increase in median scores from progress tests 2 through 4. The final year of undergraduate showed a limited increase compared to the 5th year. There is a consistent increase in the median, although with fluctuations between the observed intervals.

**Conclusion:**

The progress test promoted the establishment of regular feedback among students, teachers and coordinators and paved the road to engagement much needed to construct an institutional programmatic assessment.

## Introduction

High-quality educational programs benefit from structured assessment integrated with teaching methods. Such assessments offered regularly and with formative emphasis provide a rich course methodology. The academic environment underpins relevant teaching and learning through active participation, where students develop and refine professional behaviors. It requires structure, a well-described teaching plan with clear content, periods of reflection on one’s own learning and evaluations throughout the teaching and learning process [1].

The Progress Test is an individual assessment (IPT) given to all students at the same time and regularly throughout the academic program. It tests the expected knowledge after completing a certain level. The main purpose is the longitudinal, repeated and demonstrable measurement of knowledge acquisition [2].

Programmatic assessment aims to enhance learning and curriculum by integrating assessments and feedback cycles, treating each as a data point as well as inputs for making pass/fail decisions. It relies on a multitude of methods supported by educational purposes and instructor guidance. It must identify areas in the curriculum that require attention; promote feedback mechanisms for both students and teachers, and inform teaching processes with a focus on learning strategies [3]. It is based on formative assessment of learning, summative decisions regarding course progression, and the evaluation of the quality of the educational process, all on a regular basis. It strongly depends on institutional functions, particularly the need for centralization and standardization, integration, scope and process continuity. The methods must be accountable for learning and serve curricular purposes [4–6].

The IPT as described here has been the road to reach the engagement as described by van der Vleuten [5]. The schools which data comprise this investigation employs assessments with project reports of field practices; a by-monthly regular assessments that are electronically recorded with feedback; and a personal portfolio. This investigation presents the results of four serial applications of the progress test aimed at continuously assessing individual performance and utilizing it as an instrument to support the construction of a programmatic assessment.

## Methods

The IPT was applied to a consortium of medical schools under a common curriculum [https://www.inspirali.com/graduacao/] as a means to structure a programmatic assessment integrated into teaching. The IPT informs students, faculty and coordinators about the progress of the educational process. Students receive their scores in areas of high performance or with limitations that may require greater attention. Teachers verify topics with low performance and can, through critical reviews, better allocate teaching time and resources. Coordinators receive information about the use of educational structure and processes. The IPT is a semi-annual voluntary activity aimed at all medical students from the first to the sixth year.

The blueprint for the PT considers the Miller’s level for knowledge as recall, comprehension or analysis. Briefly, Miller’s levels refer to the development of competence into four hierarchical processes – the lower for knowledge, the second for application of knowledge, the third for clinical skills and the fourth for clinical performance – designed as a pyramid [7]. Each assessment has 120 items, all with a short stem, one direct question, and four alternatives with one correct and three distractors. The key and its feedback are provided in the database. The items refer to the curricular content weighted by frequency and impact to exercise different levels of importance [8]. The blueprint has a map of purposes – foundational elements, that align with the intended outcomes and goals to be achieved in that particular phase, a stated intentionality. These three conditions can be combined in any form or presented in isolation. The guideline also addresses the difficulty level. A committee reviews, validates and selects the items equally distributed for the classification system of the five major areas. The IPT is offered online and on site, lasting four hours and providing the number of correct answers at the end, relying on IRT analysis. The student accesses a secure platform using a login and password. A public notice describes and guides the students’ voluntary participation each semester.

Most of the items have context such as clinical case, real-life situations, experimental descriptions, scientific literature, and are aligned with a common curricular matrix. These questions were categorized using an Excel spreadsheet, and its final results were entered into an electronic platform on the website (https://www.ulife.com.br/login.aspx) to create the test.

Questions are classified using three levels. The first level pertains to five areas of the national Medical Residency exams: Internal Medicine; Surgery; Gynecology and Obstetrics; Pediatrics; Preventive and Social Medicine. The second level of classification relies on topics covered in the proficiency exam conducted by the National Board of Medical Examiners – Exam Content [https://www.nbme.org/assessment-products/assess-learn/subject-exams]. The third level of classification encompasses a flexible order that includes various medical specialties related to the national (Brazil) Unified Health System (SUS) and other miscellaneous topics, such as Bioethics; Pharmacology; Physiology; Cardiology; Neurology; Oncology; Childcare; Orthopedics; Emergency; Trauma; and Intensive therapy. The professors, whose authorship was registered, identified the curriculum origins for the topics evaluated.

The IPT uses Item Response Theory (IRT), a family of probabilistic models that places the ability levels of test-takers and the difficulty of the items on the same scale. In IRT, the likelihood of a correct answer on each item is estimated using a logistic function with the scale where items’ difficulties and ability levels of individual are situated. While IRT models can be computed with one to three parameters (difficulty, discrimination, and pseudo-guessing), we prefer using the Rasch model for operational purposes, which estimates only the difficulty parameter of each item.

In the context of the Rasch model, item fit refers to the degree to which a specific question or item on a test aligns with the overall pattern of responses from participants. A well-fitting item demonstrates an expected relationship between a participant’s ability and their likelihood of providing a particular response. On the other hand, person fit evaluates how consistently an individual’s responses conform to the expected patterns of the model. When an item or person does not fit well, it suggests a potential misalignment between the observed data and the Rasch model’s expectations, indicating that the item might be too difficult or easy for the participant’s ability level or that the participant’s responses might be inconsistent with their overall pattern of responses. A good level of item fit can be seen as validity evidence based on internal structure. The analyses were all performed through a software Xcalibre, Version 4.2.2.0 (https://assess.com/xcalibre/) in order to provide a scale from 0 to 1,000 for students’ performance.

Starting with the second edition of the IPT, ten items were consistently resubmitted: ten items from IPT1 to IPT2, ten items from IPT2 to IPT3 and ten items from IPT3 to ITP4. The inclusion criterion for these items was a higher discrimination parameter in the previous test, even though we did not apply any formal test equating procedure.

## Results

The tests were administered to students at the end of semesters during specific years: the second semester in 2020 (IPT1), the first (IPT2) and second semesters of 2021 (IPT3) and first semester of 2022 (IPT4). The participation rates were as follows: IPT1 had 23.8% (*n* = 1,055) of those enrolled students (*n* = 4,439); IPT2 had 59.5% (*n* = 3,007) of enrollees (*n* = 5,053), IPT3 had 76.6% (*n* = 7,656) of (*n* = 9,994) and IPT4 81.9% (*n* = 9,069) of (*n* = 11,073). The number of enrolled students increased from 4,439 (IPT1) to 11,073 (IPT4) due to the inclusion of new schools in the consortium. Initially, the progress tests in the first and second editions were offered to eight schools, while from the third edition onward, the number of participating institutions was expanded to 14 schools.

In all four IPT editions, representatives from all twelve semesters participated, except for the first test, which did not include students from the 12th semester. The number of participants increased in each edition, mainly because the IPT was gaining popularity among students who were not familiar with the test in the first edition. The results indicated a growing level of participation, and, with the exception of IPT1, the medians showed a slight increase from the first to the twelfth semester (Table 1). Reliability for each edition is shown (Table 2).

**Table 1:** Participants in each IPT edition and median in each semester

	TPI1		TPI2		TPI3		TPI4	
***1st***	*83*	*513.0*	*605*	*446.4*	*61*	*462.2*	*1547*	*465.7*
***2nd***	*170*	*530.0*	*187*	*433.4*	*1490*	*456.4*	*112*	*471.9*
***3rd***	*79*	*513.0*	*419*	*465.6*	*293*	*456.4*	*1404*	*482.0*
***4th***	*140*	*506.2*	*273*	*471.9*	*1293*	*490.5*	*410*	*482.0*
***5th***	*85*	*502.7*	*387*	*520.9*	*448*	*490.5*	*1289*	*505.5*
***6th***	*98*	*526.6*	*203*	*514.8*	*1186*	*517.9*	*450*	*478.0*
***7th***	*120*	*506.2*	*230*	*535.9*	*595*	*523.4*	*1143*	*520.7*
***8th***	*110*	*516.5*	*177*	*568.8*	*844*	*550.2*	*621*	*516.9*
***9th***	*61*	*509.6*	*207*	*574.8*	*341*	*544.8*	*846*	*543.1*
***10th***	*64*	*516.4*	*118*	*602.0*	*740*	*587.7*	*386*	*531.9*
***11th***	*45*	*519.9*	*113*	*642.2*	*230*	*555.5*	*718*	*573.0*
***12th***	*-*	*-*	*88*	*632.5*	*135*	*609.3*	*143*	*531.9*
***Total***	*1055*		*3007*		*7656*		*9069*	

**Table 2:** Reliability coefficients and 95% confidence intervals (95% Cis), four editions of the progress tests (IPT)

Reliability Estimate	IPT 1	IPT2	IPT 3	IPT 4
**McDonald’s ω** **(95% CI)**	0.946 (0.942–0.951)	0.923 (0.919–0.927)	0.908 (0.905–0.911)	0.926 (0.924–0.929)
**Cronbach’s α** **(95% CI)**	0.945 (0.941–0.950)	0.922 (0.918–0.926)	0.907 (0.904–0.910)	0.925 (0.923–0.927)

The boxplots (median and interquartile range) represent the performance of participants in each semester of the progress test. It is important to note that they do not represent the exact same universe of examinees, as some students did not take part in all four consecutive tests, as discussed earlier. There were modest improvements from the first semester to the final twelfth one, with the following changes:

IPT1 513 [474–560] up to 520 [438–547] (1.3%);

IPT2 446 [399–490] up to 633 [501–689] (41.7%);

IPT3 462 [420–491] up to 609 [534–660] (31.8%).

IPT4 466 [440–498] up to 532 [453–616] (14.2%).

**Fig. 1:**
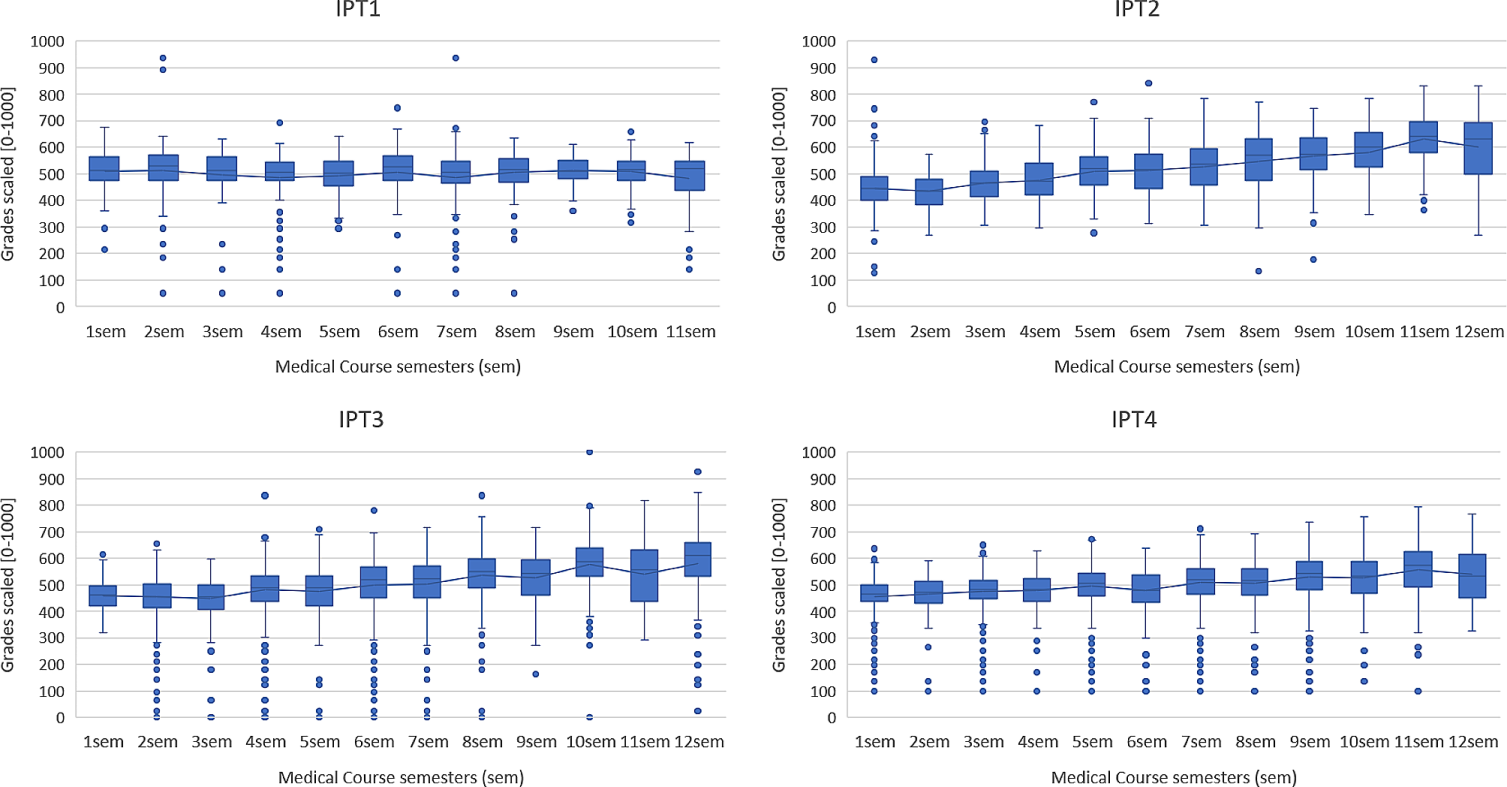
Scaled grades (boxplots) in four individual progress tests (IPT) Boxplots: minimum values (lower stem); first quartile (25%), median and third quartile (75%) – in the “box”, and highs (upper stem). Single points (“outside the curve”) are scores below or above the statistical results for minimum and maximum

There was a consistent upward trend in medians, although there were fluctuations among the semesters (Fig. 1).

**Fig. 2:**
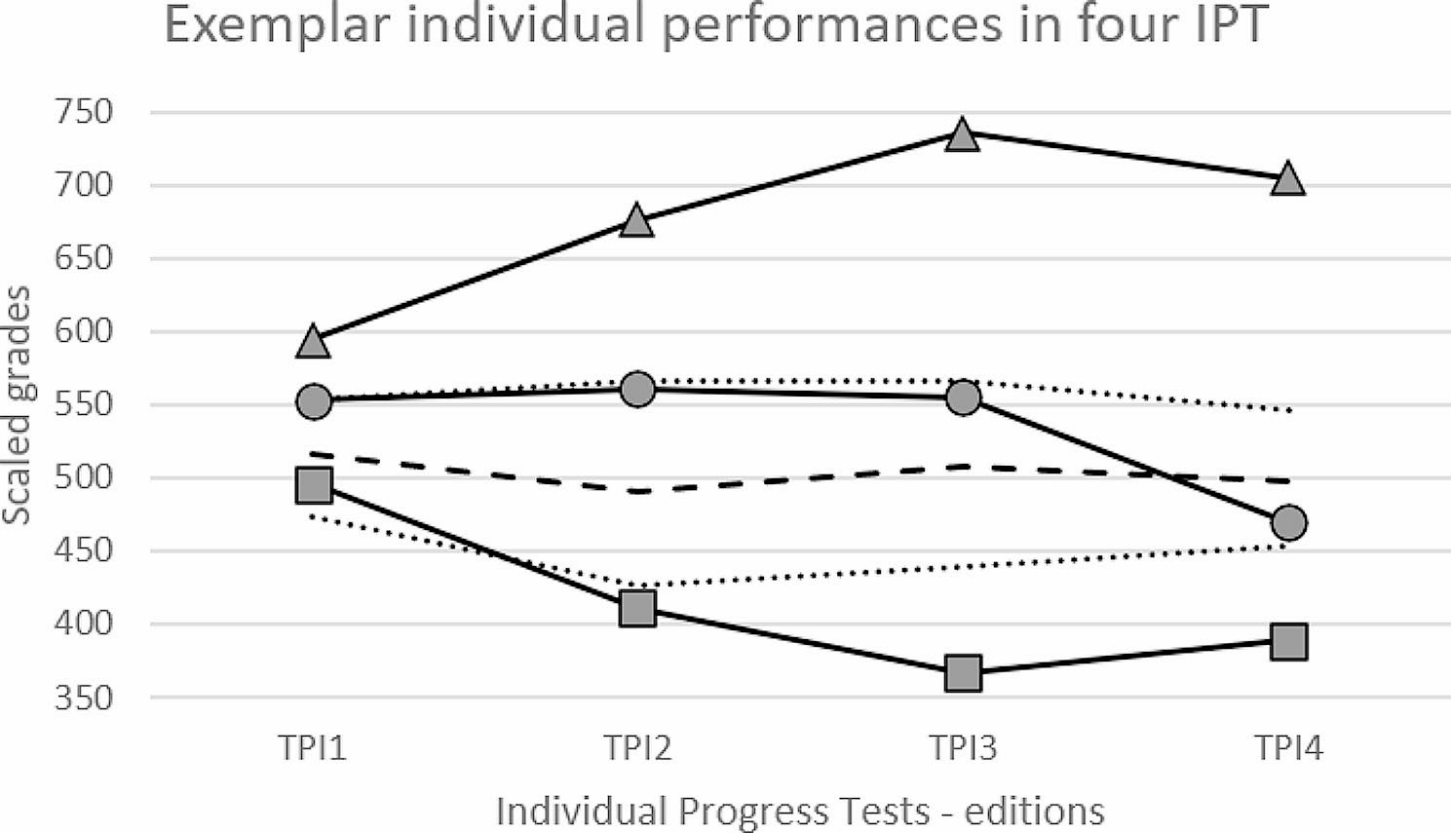
Results of three exemplar individual performances at four IPTs Median (dashed line), interquartile percentiles (dotted lines) from totaling of four IPT editions. Upward performance exemplar representations (triangle); stable (circle); descending (square)

The results enabled a follow-up of individual performances, particularly focusing on trajectories that exhibited upward, stable or downward performance trends, all of which are linked to the median and 25–75% percentiles. Examples of these behaviors, observed randomly without identification, revealed distinct patterns of results (Fig. 2). The highlighted examples demonstrate real trajectories over the course of the semesters. These three exemplars exhibited the following grades across the sequential progress tests from the first to the fourth: upward (594-676-736-705); stable (553-561-556-470) and downward (496-411-367-389). A software-driven process enables students, instructors and coordinators to extract these insights effectively. Briefly, they can select individual or collective data stats (a semester or a school, for instance) to inform a decision related to the topics in that particular period.

**Fig. 3:**
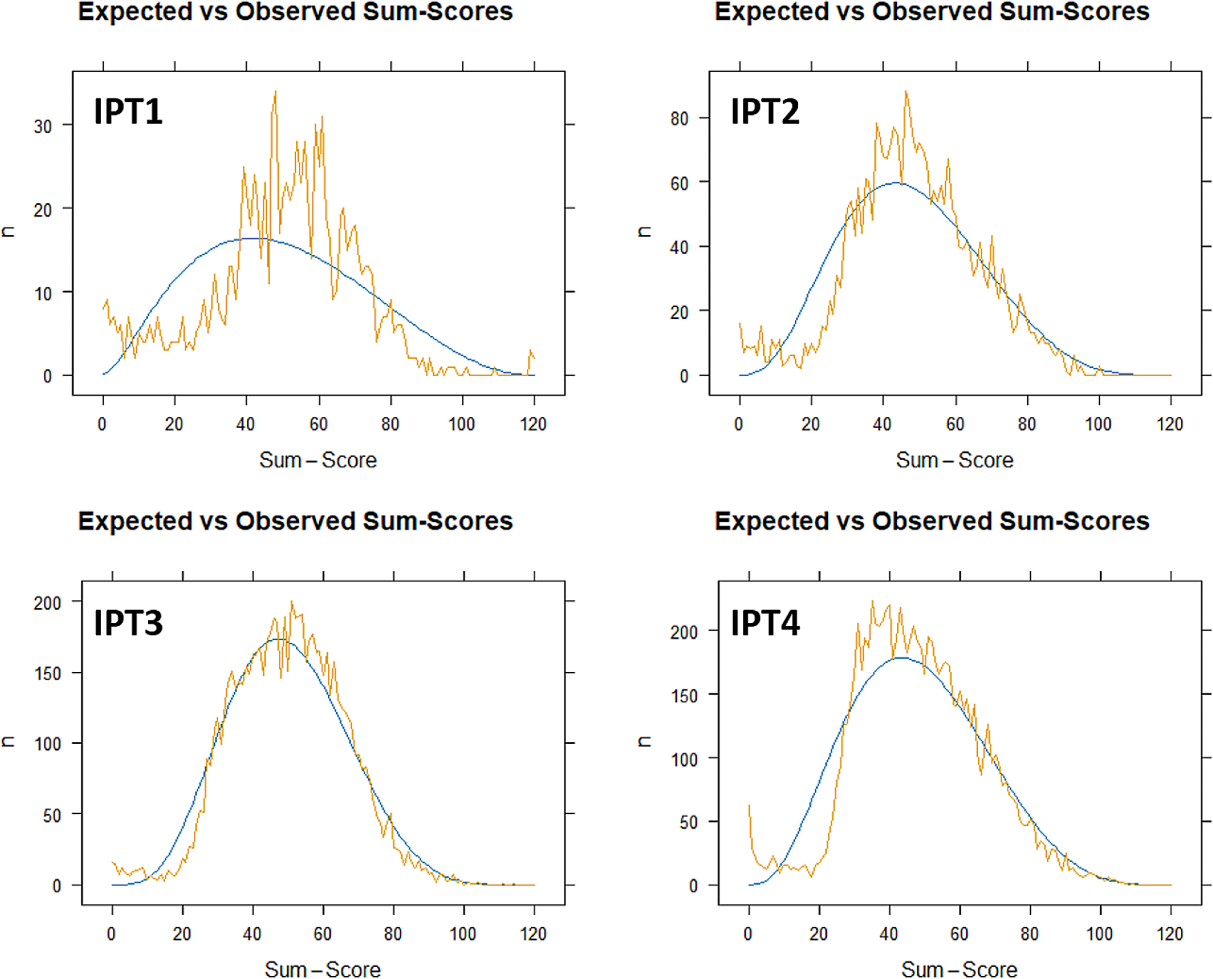
Expected and observed sum-score curves for all ITP, one to four The curves for expected (smooth) and observed (jagged) sum-score indicate a good fit for the data, except for the IPT1

The fit of the IRT model to the data indicates that only the IPT1 does not appear to be entirely accurate (Fig. 3). While this may not be the most desirable outcome, it is worth noting that given the novelty to the entire community of instructors (who prepare the items) and students (who take the IPT), this result is not significantly distant from the other IPT editions, aligning with the principles of assessment.

Finally, the content representation of the ten items resubmitted, as described in the section Methods, showed consistency from IPT1 to IPT4. In the IPT1-IPT2 the correlation for the *discrimination index* reached *r* = 0.96; for IPT2-IPT3 reached *r* = 0.83 while in IPT3-IPT4 there was a slightly flat *r*=-0.14 (negative).

## Discussion

The individual progress test (IPT) has shown improvements between subsequent semesters. These results have the potential to guide students by providing feedback on areas of strength and areas that need improvement. At the institutional level, they contribute to the evaluation of didactic, structural processes, and strategies.

The variation in the number of participants mirrors growing awareness of the test, encouragement from professors and peers, and the increased participation of more institutions from the third IPT onwards. While the number of students does vary, likely due to the non-compulsory nature of the exam, we provided three examples of individual performance to encourage participation. With the exception of the first progress test (IPT1), the results in subsequent semesters consistently showed increasing percentages, aligning with the literature in the field. This reflects a 7% increase between the 1st and 2nd years; 12% increase between the 2nd and 3rd years; and an 8% increase between the 3rd and 4th years, as well as between the 4th and 5th years of study [9].

Students receive detailed feedback in their classes, which aligns with their instructors’ areas of teaching. They get their complete test scores and sub-scores, categorized as per the blueprint. Coordinators help instructors identify areas of lower student performance to address any gaps. Additionally, personalized feedback is integrated into students’ portfolios to foster engagement and reflection [10]. This approach is consistent with institution’s responsibility for creating a cohesive evaluation program, providing data for faculty to make decisions about the summative assessments, and supporting individual needs and motivation for outstanding performance [3]. In this way, these progress tests contribute to the development of programmatic assessment [4]. The IPT items are classified and tagged to provide for reviewing according to the IRT results (discrimination index), and to align with novel items as well as with the curriculum blueprint.

The described results align with several principles of effective institutional evaluation. They demonstrate coherence through coordinated and aligned sequential individual assessments serving the same purpose, namely, evaluating performance. These evaluations exhibit continuity and comprehensiveness, as individual results contribute to curricular reviews. They are practical, offering realistic and context-based content oriented towards individual follow-up. Moreover, they are deemed acceptable and transparent, providing personalized results, allowing voluntary participation, and granting academic credits [11].

Distinguishing regular assessment from progress testing is crucial. Regular assessment provides immediate feedback, a feature not easily matched by progress tests, particularly when using item response theory. Regular testing focuses primarily on specific subjects, whereas progress tests assess cumulative knowledge. Progress tests evaluate an educational program’s effectiveness, while regular assessments likely help reinforce concepts, retention, and long-term memory. They also enable educators and administrators to assess the impact of teaching methods, instructional interventions, or curricular changes on student learning outcomes. Given its broad scope, progress tests may reveal curriculum shortcomings in preparing students, knowledge gaps, or areas of underperformance needing attention.

Individual results offer valuable guidance for students. Two key aspects are notable: (1) In cases of declining performance, instructors and mentors can intervene to support student in achieving better results; (2) When performance is on the rise, it indicates the potential for additional academic opportunities, such as monitoring programs or supplementary training. These processes pave the way for a lifelong learning, extending education throughout one’s professional career.

The online format conserves resources, enabling increased investment in evaluations via portfolios, simulations and real-world performance assessments [12]. Such an environment, fostering reflective learning through assessments as described here, forms the basis for programmatic assessment and professional development. This shared responsibility for health education starts during undergraduate studies [1].

Integrating progress testing into programmatic assessment allows for longitudinal data collection, informing program and material design to cater to learner and instructor needs. This approach can shift the focus from a “testing culture” to a “culture of evaluation,” emphasizing overall learning and development over individual assessment performance [13]. It is worth to point that in this conglomerate of medical schools there are also surveys and reviews of curriculum documents, in line with the literature of curriculum development and management [14]. 

This report has significant limitations. While the number of participants fell short of the total, nearly all students participated voluntarily. Some consortium schools are relatively new and do not offer a complete 12-semester medical course. The stable median performance has drawn criticism, possibly reflecting a curriculum in development. The progress test can inspire programmatic assessment but may not suffice for the endeavor. Equating of the tests was omitted. This decision can be justified by the unique features of the Rasch model. It provides sample-independent parameters, maintaining stable item difficulty estimates, regardless of the specific group of test-takers. The scale is anchored to the item difficulty parameters, allowing the Rasch model to estimate “true scores” for participants, irrespective of variations in test difficulty levels. Using the same scale for item difficulties and participants’ abilities inherently captures participants’ progress along a consistent scale, reducing the need for traditional test equating.

The progress tests and their reports by areas and themes promoted the establishment of regular feedback by teachers and students, as well as by teachers and course coordinators. The individual guidance on regular intervals paved the road to engagement much needed to construct an institutional programmatic assessment and better processes and strategies.

## Data Availability

All data consisting of Excel files are available on request. Please contact the correspondence author at: joaquimev@usp.br.
